# Single-Dose Tafenoquine to Prevent Relapse of *Plasmodium vivax* Malaria

**DOI:** 10.1056/NEJMoa1710775

**Published:** 2019-01-17

**Authors:** M.V.G. Lacerda, A. Llanos-Cuentas, S. Krudsood, C. Lon, D.L. Saunders, R. Mohammed, D. Yilma, D. Batista Pereira, F.E.J. Espino, R.Z. Mia, R. Chuquiyauri, F. Val, M. Casapía, W.M. Monteiro, M.A.M. Brito, M.R.F. Costa, N. Buathong, H. Noedl, E. Diro, S. Getie, K.M. Wubie, A. Abdissa, A. Zeynudin, C. Abebe, M.S. Tada, F. Brand, H.-P. Beck, B. Angus, S. Duparc, J.-P. Kleim, L.M. Kellam, V.M. Rousell, S.W. Jones, E. Hardaker, K. Mohamed, D.D. Clover, K. Fletcher, J.J. Breton, C.O. Ugwuegbulam, J.A. Green, G.C.K.W. Koh

## Abstract

**BACKGROUND:**

Treatment of *Plasmodium vivax* malaria requires the clearing of asexual parasites, but relapse can be prevented only if dormant hypnozoites are cleared from the liver (a treatment termed “radical cure”). Tafenoquine is a single-dose 8-aminoquinoline that has recently been registered for the radical cure of *P. vivax*.

**METHODS:**

This multicenter, double-blind, double-dummy, parallel group, randomized, placebo-controlled trial was conducted in Ethiopia, Peru, Brazil, Cambodia, Thailand, and the Philippines. We enrolled 522 patients with microscopically confirmed *P. vivax* infection (>100 to <100,000 parasites per microliter) and normal glucose-6-phosphate dehydrogenase (G6PD) activity (with normal activity defined as ≥70% of the median value determined at each trial site among 36 healthy male volunteers who were otherwise not involved in the trial). All patients received a 3-day course of chloroquine (total dose of 1500 mg). In addition, patients were assigned to receive a single 300-mg dose of tafenoquine on day 1 or 2 (260 patients), placebo (133 patients), or a 15-mg dose of prima-quine once daily for 14 days (129 patients). The primary outcome was the Kaplan– Meier estimated percentage of patients who were free from recurrence at 6 months, defined as *P. vivax* clearance without recurrent parasitemia.

**RESULTS:**

In the intention-to-treat population, the percentage of patients who were free from recurrence at 6 months was 62.4% in the tafenoquine group (95% confidence interval [CI], 54.9 to 69.0), 27.7% in the placebo group (95% CI, 19.6 to 36.6), and 69.6% in the primaquine group (95% CI, 60.2 to 77.1). The hazard ratio for the risk of recurrence was 0.30 (95% CI, 0.22 to 0.40) with tafenoquine as compared with placebo (P<0.001) and 0.26 (95% CI, 0.18 to 0.39) with primaquine as compared with placebo (P<0.001). Tafenoquine was associated with asymptomatic declines in hemoglobin levels, which resolved without intervention.

**CONCLUSIONS:**

Single-dose tafenoquine resulted in a significantly lower risk of *P. vivax* recurrence than placebo in patients with phenotypically normal G6PD activity. (Funded by GlaxoSmith-Kline and Medicines for Malaria Venture; DETECTIVE ClinicalTrials.gov number, NCT01376167.)

Approximately 2.5 billion people are at risk for *Plasmodium vivax* malaria.^1^ The treatment and control of *P. vivax* are complicated by a latent, undetectable form in the liver stage of the parasite lifecycle, known as the hypnozoite.^2^ Hypnozoites can cause multiple clinical relapses, which can increase the disease burden and the potential for onward transmission and impede efforts to eliminate malaria.^2^

The World Health Organization recommends treatment of *P. vivax* malaria with a blood schizonticide (chloroquine or artemisinin-based combination therapy) to clear asexual parasites, plus treatment with the 8-aminoquinoline primaquine for 14 days to kill hypnozoites and prevent relapse, a treatment termed “radical cure.”^3^ Nonadherence to the primaquine regimen jeopardizes the effectiveness of the treatment.^4-11^ Reported nonadherence rates (13.6 to 33.3%) probably underestimate the real-world situation.^4-11^ Tafenoquine is a longer-acting 8-aminoquinoline,^12^ with a half-life of approximately 15 days,^13^ and it has recently been registered with the Food and Drug Administration and Australian Therapeutic Goods Administration as a single-dose treatment for the radical cure of *P. vivax*.

Glucose-6-phosphate dehydrogenase (G6PD) deficiency is an X-linked enzymopathy, with an estimated mean prevalence of 8% across areas in which malaria is endemic.^14^ Both prima-quine and tafenoquine cause hemolysis in persons with G6PD deficiency, and G6PD testing is recommended before treatment with these agents.^15-17^

The randomized, phase 2b–3 Dose and Efficacy Trial Evaluating Chloroquine and Tafenoquine in Vivax Elimination (DETECTIVE) investigated chloroquine plus tafenoquine and chloroquine plus placebo for the radical cure of *P. vivax* malaria in participants with phenotypically normal G6PD activity. Phase 3 data are reported here; phase 2b data were reported previously.^12^ To confirm assay sensitivity, we also assessed chloroquine plus primaquine (15 mg for 14 days). A meta-analysis of the individual-level patient data in DETECTIVE phase 3 and another phase 3 trial (the Global Assessment of Tafenoquine Hemolytic Risk [GATHER]) compared the efficacy of tafenoquine with that of primaquine in preventing recurrence of *P. vivax*.^18^

## Methods

### Trial Oversight

**Figure 1:**
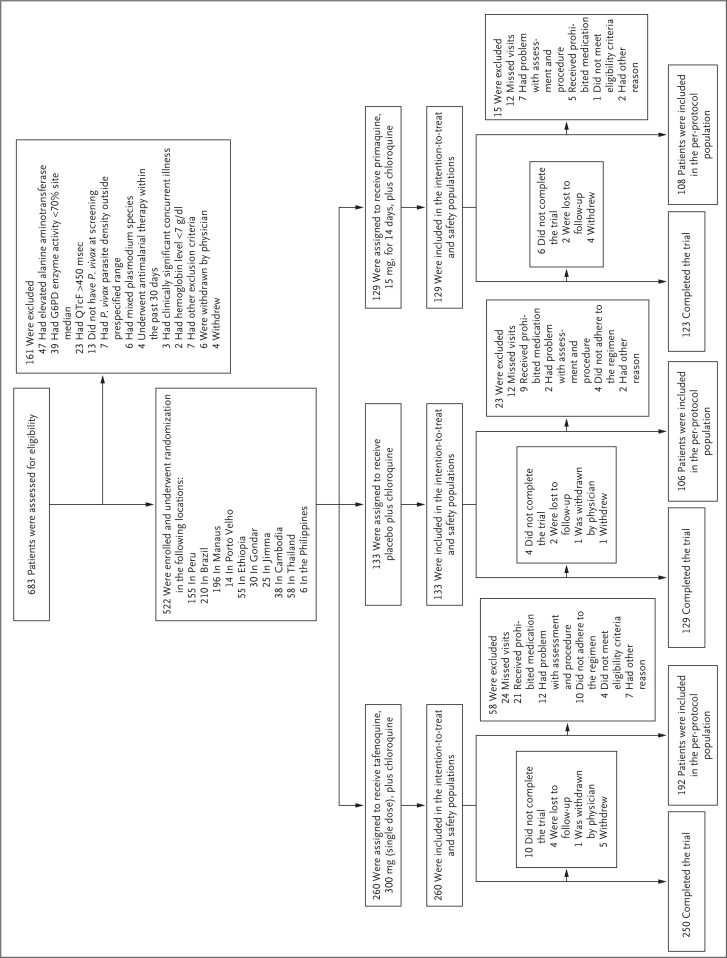
**Screening, Randomization, and Trial Populations**. The intention-to-treat population included all patients who underwent randomization and had microscopically confirmed *Plasmodium vivax* parasitemia at baseline. The per-protocol population was a subgroup of patients from the intention-to-treat population who had no major protocol violations. The safety population included all patients who underwent randomization and received at least one dose of the assigned treatment. Patients in the intention-to-treat and safety populations may have had more than one reason for exclusion. G6PD denotes glucose-6-phosphate dehydrogenase, and QTcF the QT interval corrected for heart rate according to Fridericia’s formula.

We conducted this multicenter, double-blind, double-dummy, parallel group, randomized, placebo-controlled trial from April 2014 through November 2016 at eight centers in six countries (Fig. 1). The trial was conducted in accordance with Good Clinical Practice guidelines, the principles of the Declaration of Helsinki, and relevant regulatory requirements. Ethics approval was obtained from the ethics committee or institutional review board at each site. All participants or their parents or guardians provided written informed consent. Assent was obtained from participants who were younger than 18 years of age. The protocol, including eight amendments, is available with the full text of this article at NEJM.org. The sponsors of the trial (GlaxoSmith-Kline and Medicines for Malaria Venture) contributed to the development of the protocol. GlaxoSmithKline managed the conduct of the trial, collected and managed the data, monitored the trial staff, conducted the statistical analysis, and provided all the drugs used in the trial. Medicines for Malaria Venture contributed to the design of the trial and to the analysis of the data. The first draft of the manuscript was prepared by a medical writer paid by GlaxoSmith-Kline. All authors, including those who were employees of GlaxoSmithKline, contributed to subsequent drafts, approved the final version of the manuscript, and vouch for the completeness and accuracy of the data and for the fidelity of the trial to the protocol. The sponsors and the authors agreed to maintain the confidentiality of the data during the review process.

## Participants

Eligible participants were 16 years of age or older (≥18 years of age if in Ethiopia) and had microscopically confirmed *P. vivax* infection (>100 to <100,000 asexual parasites per micro-liter) and a corrected QT interval according to Fridericia’s formula (QTcF) of less than 450 msec. Eligible female participants had negative pregnancy tests, were not lactating, and used approved contraception. Exclusion criteria were mixed plasmodium species or severe malaria, severe vomiting, a hemoglobin level of less than 7 g per deciliter, an alanine aminotransferase level more than 2 times the upper limit of the normal range, clinically significant concurrent illness, use of an antimalarial drug within the previous 30 days or use of an investigational drug within the previous 30 days or within five half-lives (whichever was longer), use of concomitant drugs that could affect trial results, previous participation in or treatment within the trial, and a history of allergies or contraindications to tafenoquine, primaquine, or chloroquine. Patients with G6PD deficiency were also excluded. Each trial site established a median value for normal G6PD activity among 36 healthy male volunteers who otherwise were not involved in the trial. Patients with G6PD activity that was less than 70% of this median, as determined on the basis of a quantitative spectrophotometric phenotype assay (Trinity Biotech), were considered to have G6PD deficiency and were excluded. Participants with a history of ocular disease or corneal or retinal abnormalities were excluded from ophthalmic evaluation.

## Randomization and Trial Regimen

The trial drugs included chloroquine phosphate (300-mg tablets [West Ward Pharmaceuticals] and 150-mg tablets [Alliance Pharmaceuticals]), tafenoquine (150-mg tablets [GlaxoSmithKline]), and primaquine phosphate (15-mg encapsulated tablets [Sanofi]) (dosages are given for the free-base form). All drugs and placebo were administered orally with food and were provided without cost to all participants.

With the use of a computer-generated randomization schedule provided by GlaxoSmithKline, on day 1 eligible participants were randomly assigned, in a 2:1:1 ratio, to receive tafenoquine, primaquine, or placebo. Patients in the tafenoquine group received 300 mg of tafenoquine in a single dose on day 1 or day 2, and patients in the primaquine group received 15 mg of prima-quine once daily for 14 days (administration was directly observed on days 1 to 3). Tafenoquine-matched and primaquine-matched placebos were administered to maintain masking. All patients also received open-label chloroquine at a dose of 600 mg on day 1 and day 2 and 300 mg on day 3; administration of chloroquine was directly observed. Patients and trial staff were unaware of the group assignments.

### Procedures

Patients remained in the clinic from day 1 to day 3 or until parasite clearance, and trial visits were scheduled on days 5, 8, 11, and 15 during the treatment period, with follow-up on days 22, 29, 60, 90, 120, 150, and 180. Insecticide-treated bed nets were provided to all patients. Patients were instructed to return to the clinic if they had symptoms of malaria; if parasitemia was found, they were given rescue therapy according to local guidelines. A list of criteria for discontinuation of therapy is provided in Table S1 in the Supplementary Appendix, available at NEJM.org.

Baseline demographic data and a medical history were obtained at screening, and a physical examination was performed. Thick and thin blood smears were prepared for identification of parasites, and parasites were enumerated with the use of established methods.^19^ Blood samples for parasitologic assessment were obtained at screening and twice daily (every 6 to 12 hours) from day 1 until day 3 or until parasite clearance was confirmed; smears were examined on all subsequent follow-up visits and after recurrence of parasitemia or withdrawal from the trial. Dried blood spots were collected on filter paper at screening and at the time of recurrence for polymerase-chain-reaction genotyping, with homology or heterology assessed with the use of published markers.^20,21^ At screening and on days 60 and 120, two measurements of G6PD enzyme levels were obtained according to the instructions of the manufacturer of the assay, and the results were averaged. G6PD genotyping was performed at a central laboratory for all female participants and for male participants with a hemoglobin decline of 3 g or more per deciliter, with the use of standard methods.^22,23^ A qualitative point-of-care test (CareStart, AccessBio) to determine G6PD activity was also performed at screening but was not used to determine eligibility. Whole-blood specimens were collected from all patients and were placed in EDTA tubes for genotype analysis of cytochrome P-450 2D6 (CYP2D6).^24,25^

Safety was assessed at all visits, at the time of recurrence of parasitemia, and at withdrawal from the trial. Twelve-lead electrocardiography was performed at screening; 12 hours after the first dose of tafenoquine, primaquine, or placebo; and on day 29. Analyses of hematologic and clinical chemistry values and urinalysis were performed at screening, on day 3, at follow-up visits until day 120, and at the time of recurrence of parasitemia or withdrawal from the trial. Methemoglobin levels were determined with the use of pulse oximetry at screening; on days 2, 3, 5, 8, 11, 15, 22, 29, 60, and 120; and at the time of recurrence of parasitemia or withdrawal from the trial. Ophthalmic assessments were performed at one site (Manaus, Brazil) at screening, on days 29 and 90, on day 180 if results were previously abnormal, and at withdrawal from the trial. Data on health outcomes and pharmacokinetic measures were recorded (findings are not reported here).

### Outcomes

The primary efficacy outcome was the percentage of patients who were free from recurrence at 6 months (termed “recurrence-free efficacy” in the protocol and Supplementary Appendix). Freedom from recurrence was defined as microscopically confirmed clearance of the initial *P. vivax* infection without recurrent *P. vivax* parasitemia and without the receipt of other antimalarial treatment during follow-up plus a negative blood smear at the 6-month assessment. Secondary efficacy outcomes were freedom from recurrence at 4 months, the time to recurrence, the time to parasite clearance (aparasitemia maintained for 6 to 12 hours), and the time to fever clearance (apyrexia maintained for 48 hours). Additional outcomes were the time to *P. vivax* gametocyte clearance (the time from the first dose until consecutive gametocyte-negative slides), *P. vivax* recurrence before day 33, the incidence of *P. falciparum* malaria, and the incidence of genetically homologous or heterologous *P. vivax*.^20^ The effect of the CYP2D6 genotype on the primary outcome was evaluated with the use of the Activity Score system, which determines a metabolic activity phenotype on the basis of the genotype (a score of 0 indicates poor CYP2D6 metabolic activity, a score of 0.5 or 1 intermediate activity, and a score ≥1.5 extensive activity).^26^ Safety outcomes were the nature and frequency of adverse events (classified according to the *Medical Dictionary for Regulatory Activities*, version 19.1), hematologic and clinical chemistry values outside the normal range, and abnormal electrocardiograms.

### Statistical Analysis

Assuming that 60% of patients in the placebo group and 90% in the tafenoquine group would be free from recurrence, we calculated that at least 90 participants would need to be enrolled in these two groups to provide the trial with more than 90% power to detect a difference between the groups. For the expanded safety database, the planned sample size was 500. Statistical analyses were performed with the use of SAS software, version 9.4 (SAS Institute).

No multiplicity adjustments were performed. The primary analysis compared the percentage of patients who were free from recurrence at 6 months in the tafenoquine group with that in the placebo group in the intention-to-treat population. Hazard ratios for the risk of recurrence of parasitemia with tafenoquine as compared with placebo and with primaquine as compared with placebo, with 95% confidence intervals and P values, were determined with the use of a Cox proportional-hazards model fitted with region and group assignment as covariates. The primary analysis was repeated for the primary outcome at 4 months, in the per-protocol population, and in a comparison of primaquine with placebo. A categorical analysis of the primary outcome in which patients with missing data were considered to have had recurrence of *P. vivax* was conducted with the use of logistic regression, with region and group assignment as covariates.

The time to recurrence of *P. vivax* malaria and clearance times for parasites, fever, and gametocytes were estimated with the use of Kaplan– Meier methods. Logistic regression was used to investigate the effect of CYP2D6 metabolism on the primary outcome in each group, after adjustment for region. Safety outcomes were summarized descriptively.

## Results

### Patients

Of the 683 patients who were screened for eligibility, 5.7% (39 patients) were excluded because they had G6PD enzyme activity that was less than 70% of the site-specific median, according to the quantitative spectrophotometric assay. The G6PD qualitative test (which was performed in 584 patients at screening) reported as “normal” all 12 patients (6 male patients and 6 female patients) who had G6PD enzyme activity that was 30% to less than 70% of the site-specific median and 4 (2 male patients and 2 female patients) of the 25 patients with G6PD enzyme activity that was less than 30% of the site-specific median. The qualitative test was not performed in 2 patients who had G6PD enzyme activity that was less than 30% of the site-specific median. Of the 522 patients who were enrolled, 502 (96.2%) completed the trial (Fig. 1). The baseline characteristics were similar across groups (Table 1). A total of 96.8% of the patients in the primaquine group (120 of 124 patients) met the prespecified criteria for adherence of at least 12 doses, as assessed by pill count and the detection of carboxyprimaquine in plasma on day 8 or 15.^27^

**Table 1:** Baseline Demographic and Clinical Characteristics in the Intention-to-Treat and Safety Populations.*

Characteristic	Tafenoquine (N = 260)	Placebo (N = 133)	Primaquine (N = 129)
Male sex — no. (%)	196 (75.4)	97 (72.9)	99 (76.7)
Age — yr	35.0±14.4	35.3±14.2	34.7±14.3
Body weight — kg			
Mean	64.9±15.9	64.1±11.2	63.1±12.3
Range	38.2–138.3	36.0–105.4	35.0–119.0
Region — no. (%)			
South America	182 (70.0)	93 (69.9)	90 (69.8)
Africa	28 (10.8)	14 (10.5)	13 (10.1)
Asia	50 (19.2)	26 (19.5)	26 (20.2)
Race or ethnic group — no. (%)†			
Multiple	97 (37.3)	47 (35.3)	47 (36.4)
American Indian	81 (31.1)	43 (32.3)	41 (31.8)
Asian	50 (19.2)	26 (19.5)	26 (20.2)
Black	28 (10.8)	14 (10.5)	13 (10.1)
White	4 (1.5)	3 (2.3)	2 (1.6)
Median *P. vivax* asexual forms (range) — no./μl	5314 (112–99,604)	5615 (101–66,010)	4380 (125–87,380)
Previous malaria — no. (%)			
Yes	219 (84.2)	106 (79.7)	109 (84.5)
No	41 (15.8)	26 (19.5)	18 (14.0)
Unknown	0	1 (0.8)	2 (1.6)
Hemoglobin (range) — g/dl			
Female patients	121.5±11.8 (96.0–154.6)	120.7±12.7 (90.0–146.0)	121.9±12.9 (83.0–146.2)
Male patients	135.8±14.2 (89.0–181.0)	133.9±13.5 (101.0–161.6)	135.7±14.6 (98.0–166.8)
G6PD enzyme level — IU/g of hemoglobin			
Mean	8.5	8.4	8.6
Range	5.6–15.5	5.8–12.0	5.4–12.5
G6PD enzyme activity — % of site-specific normal value			
Mean	103.3	102.8	104.4
Range	70.2–188.9	72.6–155.3	70.4–153.9
CYP2D6 metabolic activity phenotype — no. of patients (%)‡			
Poor	3 (1.2)	2 (1.5)	3 (2.3)
Intermediate	54 (20.8)	34 (25.6)	35 (27.1)
Extensive	192 (73.8)	94 (70.7)	87 (67.4)

* Plus–minus values are means ±SD. The intention-to-treat population included all patients who underwent randomization and had microscopically confirmed *Plasmodium vivax* parasitemia at baseline. The safety population included all patients who underwent randomization and received at least one dose of the assigned treatment. Percentages may not total 100 because of rounding. G6PD denotes glucose-6-phosphate dehydrogenase.

† Race or ethnic group was reported by the patient.

‡ Cytochrome P-450 2D6 (CYP2D6) metabolic activity was classified according to the Activity Score system, with a score of 0 indicating poor CYP2D6 metabolic activity, a score of 0.5 or 1 intermediate activity, and a score of 1.5 or more extensive activity.

### Recurrence of *P. vivax* Malaria

Five patients were considered to have had recurrence before day 33; three patients withdrew consent and therefore had incomplete day 3 assessments, one patient in the placebo group had recurrence on day 8, and one patient in the tafenoquine group had recurrence on day 19 and therapeutically inadequate plasma levels of chloroquine and desethylchloroquine.

**Figure 2:**
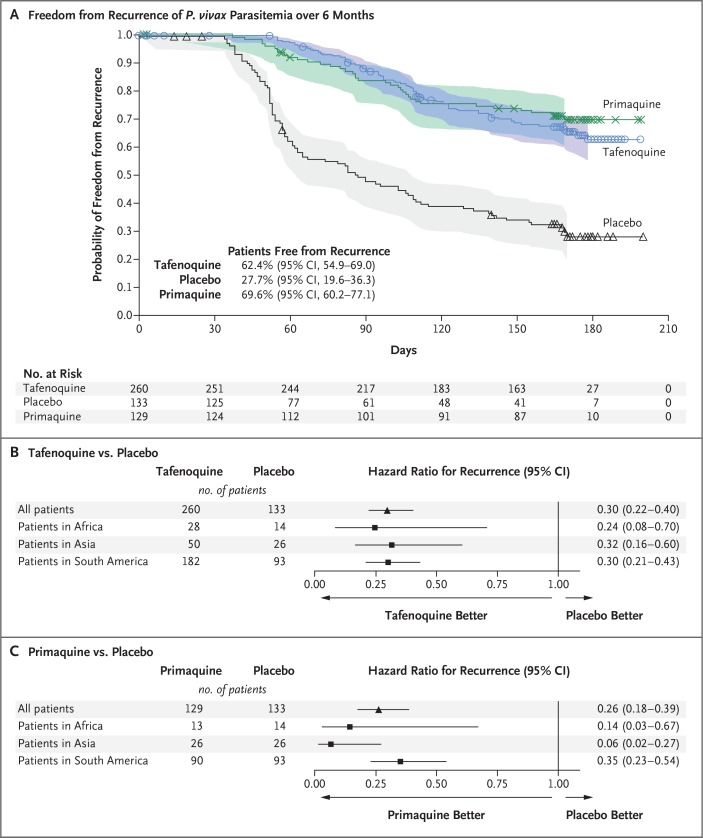
**Kaplan–Meier Analysis of the Recurrence of Parasitemia in Patients with *P. vivax***. Patients were assigned to receive tafenoquine (in a single 300-mg dose), placebo, or primaquine (15 mg, administered once daily for 14 days) in addition to a 3-day course of chloroquine (total dose of 1500 mg). Panel A shows the Kaplan–Meier analysis of the probability of freedom from recurrence of *P. vivax* parasitemia over 6 months among patients in the intention-to-treat population. The symbols indicate censored data, and the shaded areas indicate confidence intervals. Data were censored at the last parasite assessment if the patients were lost to follow-up, received a drug with antimalarial activity, or completed the trial before recurrence. Data for patients who had a recurrence before day 33 were censored at the time of recurrence. However, any patient with recurrence was considered to have had recurrence irrespective of censoring before the event. Panel B shows the hazard ratios for the risk of recurrence of parasitemia with tafenoquine as compared with placebo, and Panel C the risk of recurrence with primaquine as compared with placebo, according to region.

In the intention-to-treat population, 32.7% of the patients in the tafenoquine group (85 of 260 patients), 66.2% in the placebo group (88 of 133 patients), and 27.9% in the primaquine group (36 of 129 patients) had recurrence of *P. vivax* parasitemia at 6 months. The Kaplan–Meier estimates of the percentage of patients who were free from recurrence at 6 months were 62.4% in the tafenoquine group (95% confidence interval [CI], 54.9 to 69.0) and 27.7% in the placebo group (95% CI, 19.6 to 36.6). The hazard ratio for the risk of recurrence with tafenoquine as compared with placebo was 0.30 (95% CI, 0.22 to 0.40; P<0.001) (Fig. 2A). The percentage of patients in the primaquine group who were free from recurrence at 6 months was 69.6% (95% CI, 60.2 to 77.1). The hazard ratio for the risk of recurrence with primaquine as compared with placebo was 0.26 (95% CI, 0.18 to 0.39; P<0.001).

In an analysis in which patients with missing data were considered to have had recurrence, the results were consistent with those of the primary analysis. The percentage of patients who were free from recurrence at 6 months was 59.6% (155 of 260 patients) in the tafenoquine group, 26.3% (35 of 133 patients) in the placebo group (odds ratio for recurrence with tafenoquine vs. placebo, 0.24; 95% CI, 0.15 to 0.38; P<0.001), and 64.3% (83 of 129 patients) in the prima-quine group (odds ratio for recurrence with primaquine vs. placebo, 0.20; 95% CI, 0.12 to 0.34; P<0.001).

Analyses of the percentage of patients who were free from recurrence in the per-protocol population and at 4 months showed results that were consistent with those of the primary analysis. Findings in the tafenoquine group were similar across geographic regions (Fig. 2B). In the primaquine group, the percentage of patients who were free from recurrence was higher in Asia than in other regions, but the number of patients in Asia was small (26 patients) (Fig. 2C). The proportion of recurrences caused by parasites that were heterologous or homologous to the initial infection was similar in each of the three groups.

In patients with poor CYP2D6 metabolic activity, recurrences occurred in 1 of 3 patients in the tafenoquine group, in 2 of 2 patients in the placebo group, and in 3 of 3 patients in the primaquine group. In the tafenoquine group, there was no significant difference in recurrences between patients with poor or intermediate activity and patients with extensive activity (37.5% [21 of 56 patients] vs. 36.0% [63 of 175 patients] had recurrence; odds ratio, 1.05). In the primaquine group, patients who had poor or intermediate activity had significantly more recurrences than patients with extensive activity (38.9% [14 of 36 patients] vs. 27.5% [22 of 80 patients] had recurrence; odds ratio, 2.36). A post hoc analysis indicated that body weight had no effect on the risk of recurrence.

Parasite clearance was achieved by day 3 in 88.1% of the patients in the tafenoquine group (229 of 260 patients), in 82.7% in the placebo group (110 of 133 patients), and in 83.7% in the primaquine group (108 of 129 patients). There was no significant difference among groups in clearance times of parasites, fever, or gametocytes. *P. falciparum* infections were detected in 2.1% of all patients (11 of 522 patients) at follow-up, with no significant difference among groups. Additional information regarding recurrence is provided in Tables S2 through S10 and Figure S1 in the Supplementary Appendix.

### Safety

Adverse events of any cause were reported in a similar proportion of patients in each group (Table 2). There was no evidence that tafenoquine exacerbated the known adverse effects of chloroquine. Dizziness and decreased hemoglobin level were more common in the tafenoquine group than in the placebo group. In the tafenoquine group, 97.0% of adverse events (159 of 164 events) were mild to moderate in severity. Of the 260 patients in the tafenoquine group, 7 met the protocol-defined hematologic stopping criteria; of the 133 patients in the placebo group, 3 met the corrected QT stopping criteria. No adverse event led to withdrawal from the trial, and all events resolved without sequelae.

**Table 2:** Adverse Events in the Safety Population.*

Event	Tafenoquine (N = 260)	Placebo (N = 133)	Primaquine (N = 129)
	*number of patients (percent)*		
Any adverse event†	127 (48.8)	65 (48.9)	60 (46.5)
Pruritus	29 (11.2)	17 (12.8)	14 (10.9)
Dizziness	22 (8.5)	4 (3.0)	8 (6.2)
Nausea	16 (6.2)	9 (6.8)	7 (5.4)
Vomiting	15 (5.8)	7 (5.3)	9 (7.0)
Hemoglobin decreased	14 (5.4)	2 (1.5)	2 (1.6)
Headache	12 (4.6)	9 (6.8)	5 (3.9)
Diarrhea	10 (3.8)	4 (3.0)	3 (2.3)
Upper abdominal pain	8 (3.1)	9 (6.8)	6 (4.7)
Alanine aminotransferase increased	6 (2.3)	6 (4.5)	3 (2.3)
Serious adverse event‡	21 (8.1)	6 (4.5)	4 (3.1)
Hemoglobin decreased§	14 (5.4)	2 (1.5)	2 (1.6)
Diarrhea	1 (0.4)	0	1 (0.8)
Drug-induced liver injury¶	1 (0.4)	0	0
Hepatitis E	1 (0.4)	0	0
Limb abscess	1 (0.4)	0	0
Menorrhagia	1 (0.4)	0	0
Spontaneous abortion	1 (0.4)	0	0
Urinary tract infection	1 (0.4)	0	0
Prolongation of QTcF	0	3 (2.3)	0
Gastroenteritis	0	1 (0.8)	0
Dehydration	0	0	1 (0.8)
Nausea	0	0	1 (0.8)
Vomiting	0	0	1 (0.8)

* Shown are the most common adverse events of any cause that occurred from baseline through day 29 and all severe adverse events of any cause that occurred during the 6-month trial period. Safety data through day 29 are presented to avoid the potential confounding effects of the higher frequency of malaria recurrence and administration of rescue therapy in the placebo (chloroquine only) group. Details of adverse events occurring at any point during the trial period and severity grades are provided in Table S11 in the Supplementary Appendix. Adverse events were classified according to the *Medical Dictionary for Regulatory Activities*, version 19.1.

† Shown are adverse events occurring in more than 5% of patients in any group.

‡ Serious adverse events related to the trial regimen were prolongation of the QT interval corrected for heart rate according to Fridericia’s formula (QTcF) in three patients in the placebo group, decreased hemoglobin in one patient in the placebo group, and nausea in one patient in the primaquine group. No serious adverse events were attributed to tafenoquine.

§ Shown are decreases in hemoglobin level of more than 3 g per deciliter or 30% or more from baseline. No patient had a hemoglobin level less than 6 g per deciliter. Further details on these patients are provided in Table S13 in the Supplementary Appendix.

¶ This case of drug-induced liver injury was attributed by the investigators, who were unaware of group assignment, to unauthorized use of herbal medication.

**Figure 3:**
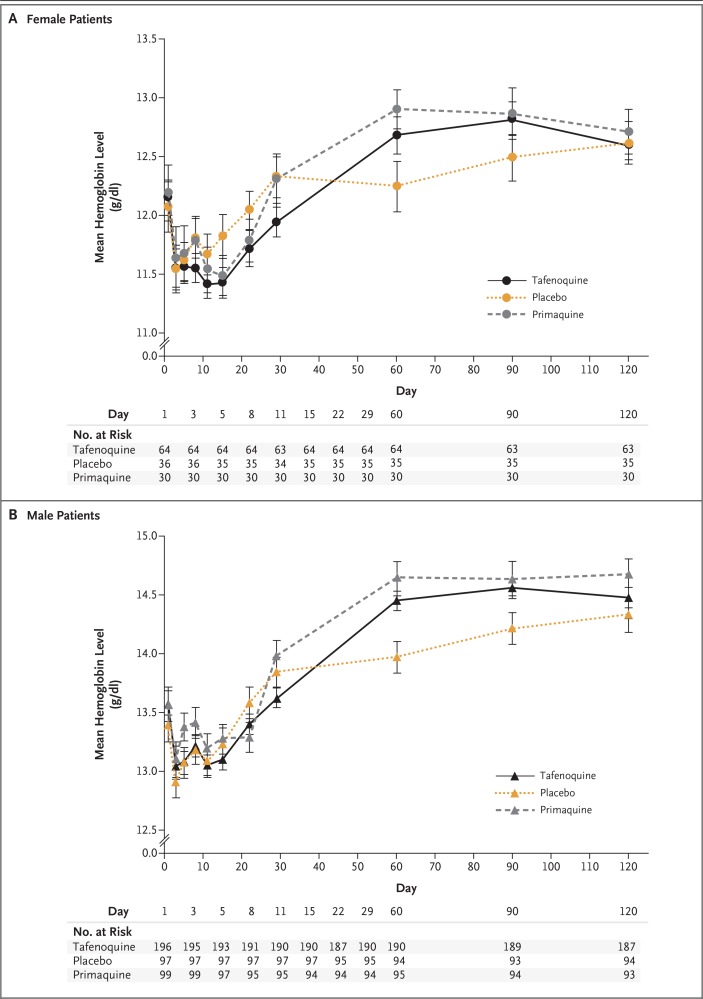
**Mean Hemoglobin Levels in Patients with Normal G6PD Genotype and *P. vivax* Parasitemia**. The y axis has been shifted upward in Panel B to allow data to be compared more easily. The number at risk indicates the number of patients who could be evaluated at each time point. I bars indicate standard errors.

A mild decline from baseline in mean hemoglobin levels was observed in all groups initially, but levels subsequently returned to the normal range (Fig. 3). Results for hematocrit were consistent with the observed changes in hemoglobin levels. Hemoglobin declines of more than 3 g per deciliter or at least 30% of the baseline level were noted in 5.4% of the patients in the tafenoquine group (14 of 260 patients), in 1.5% in the placebo group (2 of 133 patients), and in 1.6% in the primaquine group (2 of 129 patients); all these patients had a normal G6PD genotype. No patients had symptoms of anemia, and hemoglobin levels returned to the normal range without clinical intervention. Two female patients were heterozygous for a variant associated with G6PD deficiency (the *Viangchan* variant): 1 in the tafenoquine group, who had a G6PD enzyme level of 5.57 IU per gram of hemoglobin, and 1 in the primaquine group, who had a G6PD enzyme level of 5.41 IU per gram of hemoglobin; neither patient had clinically important declines in hemoglobin level (declines of 2.3 g per deciliter and 1.8 g per deciliter).

From baseline through day 29, methemoglobin levels were at least 10% in 1.2% of the patients in the tafenoquine group (3 of 260 patients), in 0% in the placebo group (0 of 133 patients), and in 7.8% in the primaquine group (10 of 129 patients). Maximum methemoglobin levels were 13.0% in the tafenoquine group and 14.7% in the primaquine group. No clinical signs of methemoglobinemia were observed. There were no differences among the groups in other hemato-logic or laboratory outcomes.

Coadministration of tafenoquine or prima-quine with chloroquine did not exacerbate the QTcF prolongation that is known to occur with chloroquine use. Ophthalmic examinations showed that in the tafenoquine group, 1 of 65 patients had unilateral keratopathy, and 2 of 65 had unilateral retinal changes; in the prima-quine group, 1 of 30 patients had unilateral retinal hypopigmentation. Postbaseline abnormalities in Humphrey visual fields were noted in 5 of 103 patients (2 of 54 patients in the tafenoquine group, 1 of 24 in the placebo group, and 2 of 25 in the primaquine group). No findings were clinically problematic or suggested an ocular effect of drug treatments. Additional information regarding safety is provided in the Supplementary Appendix.

Discussion The trial population was composed of persons living in regions in which *P. vivax* is endemic, which made them vulnerable to reinfection throughout the trial. In this population, the percentage of patients in the tafenoquine group who were free from recurrence at 6 months (62.4%; 95% CI, 54.9 to 69.0) was lower than in DETECTIVE phase 2b (89.2%; 95% CI, 77 to 95)^12^; the percentage of patients in the primaquine group and in the placebo group who were free from recurrence was also lower in this phase than in phase 2b. This could have resulted from variations in drug efficacy among trial populations, the use of different trial sites, or geotemporal differences in transmission. The larger difference between the percentage of patients who were free from recurrence at 4 months and at 6 months in this phase of the trial than in phase 2b suggests that reinfection rates were higher in phase 3 than in the previous phase. Both phases showed a consistent benefit with 8-aminoquinolines as compared with placebo.^12^ The efficacy and safety findings related to primaquine were consistent with those in other reports.^8^

Chloroquine resistance was confirmed in the case of one early recurrence and has been reported to occur in the geographic regions included the trial.^28-30^ A phase 3 trial is assessing tafenoquine plus dihydroartemisinin–piperaquine as the blood schizonticide (ClinicalTrials.gov number, NCT02802501).

The *CYP2D6* gene has high allelic heterogeneity, with wide variations in enzyme levels and activity.^31^ Tafenoquine is metabolized slowly, with no major metabolites in human plasma or urine. No effect on the efficacy of tafenoquine in preventing recurrence was seen in patients with intermediate CYP2D6 metabolic activity — a finding that is consistent with a previous analysis^25^; however, more data on persons with poor CYP2D6 metabolic activity are required. In contrast, the CYP2D6 enzyme is necessary to convert primaquine to active metabolites,^32^ and evidence is emerging that CYP2D6 metabolism can modify primaquine efficacy.^24,33,34^

Reductions in hemoglobin levels were observed in all groups and were probably the result of malaria infection. However, the reductions in hemoglobin level in the tafenoquine group (though mild) were greater than those in the placebo group. These observations are consistent with the reductions of 0.4 to 1.2 g per deciliter observed in a study conducted among healthy volunteers with normal G6PD genotypes who received 300 mg of tafenoquine.^12^ Overall, declines in hemoglobin of more than 3 g per deciliter or at least 30% of baseline levels were uncommon and occurred in patients with normal G6PD genotypes; none required clinical intervention. The two female patients who were heterozygous for the *G6PD Viangchan* variant were treated with 8-aminoquinolines, and neither one had adverse hematologic events.

Patients with G6PD deficiency who are treated with 8-aminoquinolines have a risk of severe hemolysis.^17^ G6PD testing is recommended before administration of the primaquine regimen used in this trial, and it will be required before administration of tafenoquine.^35^ The qualitative test evaluated here failed to identify 16 patients most at risk for hemolysis. If tafenoquine use is expanded, adoption of reliable quantitative point-of-care G6PD tests will be needed; such tests are not currently available but are in development.^36^

It was a challenge to reach the target recruitment number in this trial, though the trial had sufficient statistical power to assess the primary outcome. A major limitation of the trial was that *P. vivax* genotyping cannot differentiate among recrudescence, relapse, and reinfection; all may appear homologous or heterologous to the initial infection.^37^ Although this trial was conducted in regions where tropical-type *P. vivax* strains prevail (with relapses occurring 17 to 45 days after the initial infection^6^), it is possible that not all relapses were observed. Nevertheless, the risk of *P. vivax* recurrence from any cause was about 70% lower with tafenoquine than with placebo over the 6-month trial period. Given the need to reduce the global burden of *P. vivax* malaria, GlaxoSmithKline will make tafenoquine available at an affordable price in countries in which malaria is endemic to improve access to those who need it most.^38^

## Notes

Material has been reviewed by the Walter Reed Army Institute of Research, which had no objection to its presentation or publication. The opinions and assertions herein are the private views of the authors and are not to be construed as official or as reflecting the true views of the Department of the Army or the Department of Defense. The investigators have adhered to the policies of the Department of the Army for the protection of human research subjects as prescribed in regulation AR 70-25.

A data sharing statement provided by the authors is available with the full text of this article at NEJM.org.

Supported by GlaxoSmithKline (GSK) and Medicines for Malaria Venture.

Dr. Llanos-Cuentas reports receiving grant support and fees from GSK for conducting the tafenoquine clinical program; Dr. Noedl, receiving grant support and fees from GSK for clinical trial site development; Dr. Kleim, Mrs. Rousell, Dr. Hardaker, Ms. Kellam, Dr. Jones, Ms. Mohamed, Ms. Fletcher, Mr. Breton, Dr. Ugwuegbulam, Dr. Green, and Dr. Koh, being employed by and holding stocks and shares in GSK; and Ms. Clover, being employed by GSK. No other potential conflict of interest relevant to this article was reported.

Disclosure forms provided by the authors are available with the full text of this article at NEJM.org.

We thank the patients; the trial staff (Sonia Torres, Alexander Tenorio, Ricardo Panduro, Piere Arevalo, Cesar Banda, Silvia Marin, Jorge Parraguez [Iquitos, Peru]; Mônica Santos [Manaus, Brazil]; Juan Miguel Villalobos Salcedo, Roselene Ruffato, Eduardo Garbin, and the staff at Secretaria de Estado de Saude de Rondônia, Fiocruz-Rondonia [Porto Velho, Brazil]; Polrat Wilairatana, Sant Muangnoicharoen, Noppadol Siritanaratkul [Bangkok, Thailand]; Mengchuor Char, Rekol Huy, Heng Kimyi, Mariusz Wojnarski, Worachet Kuntawunginn, Oung Pheaktra, Soklyda Chann, Somethy Sok, Thay Kheangheng, Hom Sohei, Chandara Sok, Sokna Ly, Mok My, Nareth Kong, Yom You, and the staff at the Cambodia National Malaria Control Program [Anlong Veng, Oddar Meanchey, Cambodia]; Jonas Cura, Wilma Palay, Krystell Kay Edica, Ronalyn Manlongat [Palawan, Philippines]; Workagegnehu Hailu, Girum Bayissa, Helina Fikre, Addisu Ambaw, Yonael Mulatu, Aberham Abere, Mekibib Kassa, Meseret Birhane, Ligabaw Worku [Gondar, Ethiopia]; Zeleke Alemu, Elsah Tegene, Wondimagegn Adissu, Abdurehman Eshete, Zerihun Befekadu, Fetiya Bahiru, Eshetu Mulisa, Sadikalmahdi Hussen, Abonesh Taye, Kiya Kedir, Bizuwarek Sharew, Fuad Abanura, Negash Belete, Kulane Olane, Mubarik Taju, Sebleworek Negash, Menbera Fekadu, Adnan Endris, Endalkachaw Asrat, Halima Ababor, Hirut Goba, Birtikun Silito, Fekadu Jiru, Berhanu Belay [Jimma, Ethiopia]); Alison Webster (GSK clinical); Deborah Kelly, Jessica Ackert, and Renata Buckley (GSK safety); Richard Ward and Terry Ernest (GSK manufacturing and supply); Nick Carter, Andrew Mastromarino, Jenny Scott, Thomas Drury, and Chris Nystrom (GSK statistics and programming); Navinkumar Goyal (GSK clinical pharmacology); Maxine Taylor (GSK drug disposition); Robert Stocken and Christian Baumann (GSK regulatory); Maggie Lynch (GSK project management); Isabelle Borghini-Fuhrer and Wiweka Kaszubska (Medicines for Malaria Venture); Janice Culpepper (Bill and Melinda Gates Foundation);

Jeniffer Kigera, Godfrey Nyakaya, Natasha Dsouza, Niranjan G. Kulkarni, Nushara Nidhipaichit, Tanyada Yongchaitrakul, Filippi Bossatto, Carolina Seixas, Vivian Aboud, Evelyn Eder, and Rafael Paletta (GSK) and Tasaneeya Supaperm, Ruethai Wongchai, Salisa Pohpeera, Numfon Russamesuk, Carmen Rojas Vidal, Juan Vargas Valladares, Jesus Ramirez Morales, Iñes Antonio, Luzelle Raguero Tolarbas, and Murtaza Shipchandler (Pharmaceutical Product Development) for in-country trial management and monitoring; Alex Lowe (Fishawack Indicia) for administrative support for manuscript review; and Naomi Richardson (Magenta Communications) for developing the first draft of the manuscript, collating author contributions into subsequent drafts, and providing editorial and graphic support.

## Appendix

The authors’ full names and academic degrees are as follows: Marcus V.G. Lacerda, M.D., Alejandro Llanos-Cuentas, M.D., Srivicha Krudsood, M.D., Chanthap Lon, M.D., David L. Saunders, M.D., Rezika Mohammed, M.D., Daniel Yilma, M.D., Dhelio Batista Pereira, M.D., Fe E.J. Espino, M.D., Reginaldo Z. Mia, M.D., Raul Chuquiyauri, M.D., Fernando Val, Ph.D., Martín Casapía, M.D., Wuelton M. Monteiro, Ph.D., Marcelo A.M. Brito, M.Sc., Mônica R.F. Costa, M.Sc., Nillawan Buathong, M.Sc., Harald Noedl, M.D., Ermias Diro, M.D., Sisay Getie, M.Sc., Kalehiwot M. Wubie, M.P.H., Alemseged Abdissa, Ph.D., Ahmed Zeynudin, M.Sc., Cherinet Abebe, M.D., Mauro S. Tada, M.D., Françoise Brand, M.Sc., Hans-Peter Beck, Ph.D., Brian Angus, M.D., Stephan Duparc, M.D., Jörg-Peter Kleim, Ph.D., Lynda M. Kellam, M.Sc., Victoria M. Rousell, B.Sc., Siôn W. Jones, Ph.D., Elizabeth Hardaker, M.D., Khadeeja Mohamed, M.Sc., Donna D. Clover, B.Sc., Kim Fletcher, B.A., John J. Breton, M.C.M., Cletus O. Ugwuegbulam, Ph.D., Justin A. Green, M.D., and Gavin C.K.W. Koh, Ph.D.

The authors’ affiliations are as follows: Fundação de Medicina Tropical Dr Heitor Vieira Dourado, Manaus (M.V.G.L., F.V., W.M.M., M.A.M.B., M.R.F.C.), Fundação Oswaldo Cruz, Manguinhos, Rio de Janeiro (M.V.G.L.), and Centro de Pesquisa em Medicina Tropical Rondônia, Porto Velho (D.B.P., M.S.T.) — all in Brazil; Universidad Peruana Cayetano Heredia, Lima, Peru (A.L.-C., R.C., M.C.); Mahidol University (S.K.) and the Armed Forces Research Institute of Medical Sciences (C.L., D.L.S., N.B.), Bangkok, Thailand; the University of Gondar, Gondar (R.M., E.D., S.G., K.M.W.), and Jimma University, Jimma (D.Y., A.A., A.Z., C.A.) — both in Ethiopia; Research Institute for Tropical Medicine, Manila (F.E.J.E.), and Rio Tuba Nickel Foundation Hospital, Palawan (R.Z.M.) — both in the Philippines; Medical University of Vienna, Vienna (H.N.); Swiss Tropical and Public Health Institute and University of Basel, Basel (F.B., H.-P.B.), and Medicines for Malaria Venture, Geneva (S.D.) — both in Switzerland; Oxford University, Oxford (B.A.), and GlaxoSmith-Kline, Stockley Park West (J.-P.K., L.M.K., V.M.R., S.W.J., E.H., K.M., D.D.C., K.F., C.O.U., J.A.G., G.C.K.W.K.) — both in the United Kingdom; and GlaxoSmithKline, Collegeville, PA (J.J.B.).
